# Nanofiltration and microfiltration for the removal of chromium, total dissolved solids, and sulfate from water

**DOI:** 10.1016/j.mex.2019.03.012

**Published:** 2019-03-18

**Authors:** Ghasem Zolfaghari, Mehdi Kargar

**Affiliations:** Department of Environmental Sciences and Engineering, Faculty of Environmental Sciences, Hakim Sabzevari University, Razavi Khorasan, Sabzevar, P.O. Box: 397, Iran

**Keywords:** Fabrication of a hybrid system of nanofiltration (NF) and microfiltration (MF), Measuring of pollutant by Atomic Absorption Spectrometer (AAS) and UV–vis Array Spectrophotometer, Nanofiltration, Sand and carbon filters, Sulfate, Hexavalent chromium

## Abstract

This study was designed to evaluate the hybrid system performance of nanofiltration (NF) and microfiltration (MF) processes in removing the hexavalent chromium (Cr*(VI)*) and sulfate from water. To do so, we made a hybrid pilot, including 1 μm and 5 μm filters, sand filter, activated carbon filters, and a nanofilter. We studied the effects of various parameters on the removal of Cr*(VI)* from polluted water and drinking water such as pH, pressure, concentrations of chromium, concentrations of sulfate, and total dissolved solids (TDS). The selected parameters were as follows: pressure: 0.1-0.4 MPa, pH: 2–10, Cr*(VI)* concentration: 0.1–0.4 mg/l, and sulfate concentration: 40–500 mg/l. According to the results, the efficiency of chromium removal increased with increasing the pH, while increasing the pressure from 0.1 to 0.4 MPa decreased the removal rate of chromium. In addition, increasing the concentrations of sulfate led to a decreasing trend in the removal efficiency. According to the findings of the study, the hybrid pilot made is able to reduce the chromium and sulfate to the levels under the WHO standard (Cr*(VI)* = 0.05 mg/l and sulfate = 500 mg/l).

• The optimal conditions for removal of Cr*(VI)* included the initial chromium concentration of 0.1 mg/l, pressure of 0.1 Mpa, pH of 10, and the sulfate concentration of 40 mg/l.
• In general, the experimental results revealed that the fabricated hybrid system including MF, NF, sand filter, and carbon filter has the ability to remove chromium and sulfate from drinking water (tap water) at a rate of 99%.
• At sulfate concentration of 40 mg/l, the TDS elimination efficiency was 97.75% and increased by 99.87% as the concentration increased to 500 mg/l. The presence of sulfate ions increases the TDS in water.

## Graphical abstract

**Figure:**
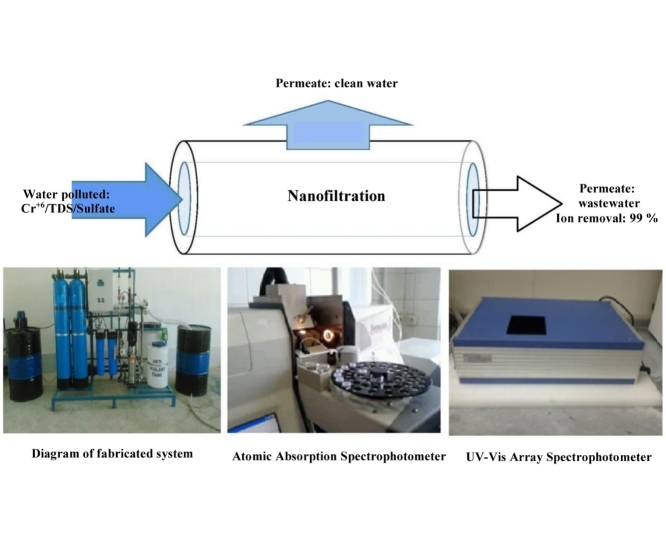

**Specifications Table**

**Table:** 

**Subject Area:**	• *Environmental Science*
**More specific subject area:**	• *water and wastewater treatment*
**Method name:**	• *Fabrication of a hybrid system of nanofiltration (NF) and microfiltration (MF)* • *Measuring of pollutant by Atomic Absorption Spectrometer (AAS) and UV-Vis Array Spectrophotometer*

## Methods details

### Analysis method

The concentrations of chromium (Cr*(VI)*) were analyzed in the feed solution under different pH conditions with an advanced Atomic Absorption Spectrometer (AAS), Contraa-700 Model. Furthermore, the concentration of sulfate anion (Sigma-Aldrich) in the samples was analyzed with a UV–vis Array Spectrophotometer (Photonix Ar 2015, Teifsanje Pishro Pajohesh Company, Iran). The pH was adjusted using NaOH and HCl. The chemicals used in this study (K_2_Cr_3_O_7_, NaOH, Na_2_SO_4,_ and HCl) were purchased from Merck Co. with a purity percentage of more than 99.99%. Chromium solution was prepared using distilled water. Many samples from tap water were tested and analyzed as well. Experiments in the laboratory temperature (25 °C) were conducted.

### Design and fabrication

**Fig. 1:**
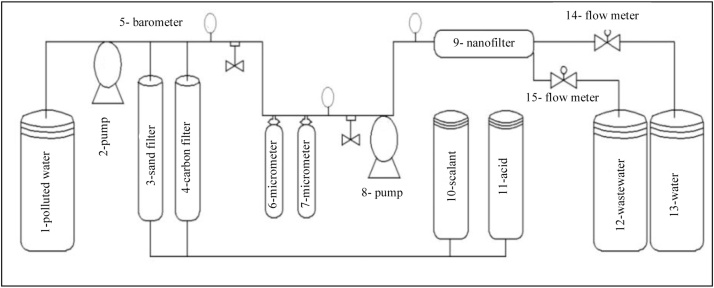
Diagram of fabricated instrument, hybrid system of nanofiltration, microfiltration, sand filter, and activated carbon filter. 1: polluted water tank, 2: pump, 3: sand filter, 4: activated carbon filter, 5: barometer, 6: microfilter (polypropylene 5 μm), 7: microfilter (polypropylene 1 μm), 8: high-pressure pump, 9: nanofilter 90–400, 10: anti scalant tank, 11: acid wash tank, 12: wastewater tank, 13: cleaned water tank 14: water flow meter, and 15: wastewater flow meter.

All the tests were performed at a pilot scale, a hybrid membrane unit made of a polyamide spiral membrane of 90–400 nanofilter type. Fig. 1 shows the schematic diagram of the pilot hybrid (microfiltration (MF) and nanofiltration (NF)) used in this study, which contains two pumps, a low-pressure feed pump connected to the sand filter, activated carbon filters, two 1 and 5 μms filters, and a high-pressure feed pump connected to the NF membrane. The hybrid pilot is equipped with sampling valves, pressure gauges, and water production and wastewater production flow meters. The water purification pilot was built at Hakim Sabzevari University and experiments were conducted at the Environmental Lab of University of Birjand.

### Filtration experiments

The experiments were begun after fully washing of the hybrid pilot. The Cr*(VI)* solution was prepared according to the concentration of examined feed in a 110-liter feed tank, which entered the 1 and 5 μm filters after passing through a sand filter and an activated carbon filter tank. Water flow through the reservoirs of sand filter and activated carbon filter respectively leads to the removal of total dissolved solids (TDS) and the elimination of possible free chlorine in the water. Then, water flow enters the high-pressure pump, which pressure is increased to the operational pressure, and finally enters the NF membrane. A pressure gauge is embedded before the activated carbon filter and after 1- and 5-micron filters to measure the pressure; in case of dropping too much, the filters had to be replaced. After passing the water containing hexavalent chromium from embedded filters, its turbidity and suspended solids are sufficiently filtered to be prepared to cross the membrane. The pressure gauges are installed in the path of input and output of the NF membrane to indicate the pressure drop, suggesting its blockage. In such an event, the membrane should be washed. A flow meter is installed in the path of the incoming water and the concentrated water to measure the feed flow rates and the penetrating water. Different levels of performing the tests are presented in Table 1. This study considers four factors, and each factor has four levels. Each experiment is repeated 3 times. A total of 120 experiments were conducted.

**Table 1:** Test conditions.

Sulfate concentration (mg/l)	pH	Pressure (bar)	Cr concentration (mg/l)	
			Real water	Distilled water
40	4	1	0.148	0.1
100	6	2	0.248	0.2
400	8	3	0.348	0.3
500	10	4	0.448	0.4

## Results and discussion

Removal of hexavalent chromium in hybrid processes were discussed in the following sections under different operating conditions such as pressure, initial Cr concentration, pH, total dissolved solids, and the concentration of sulfate.

### Chromium removal efficiency by the fabricated hybrid system

#### The effect of hexavalent chromium concentration on removal efficiency

**Fig. 2:**
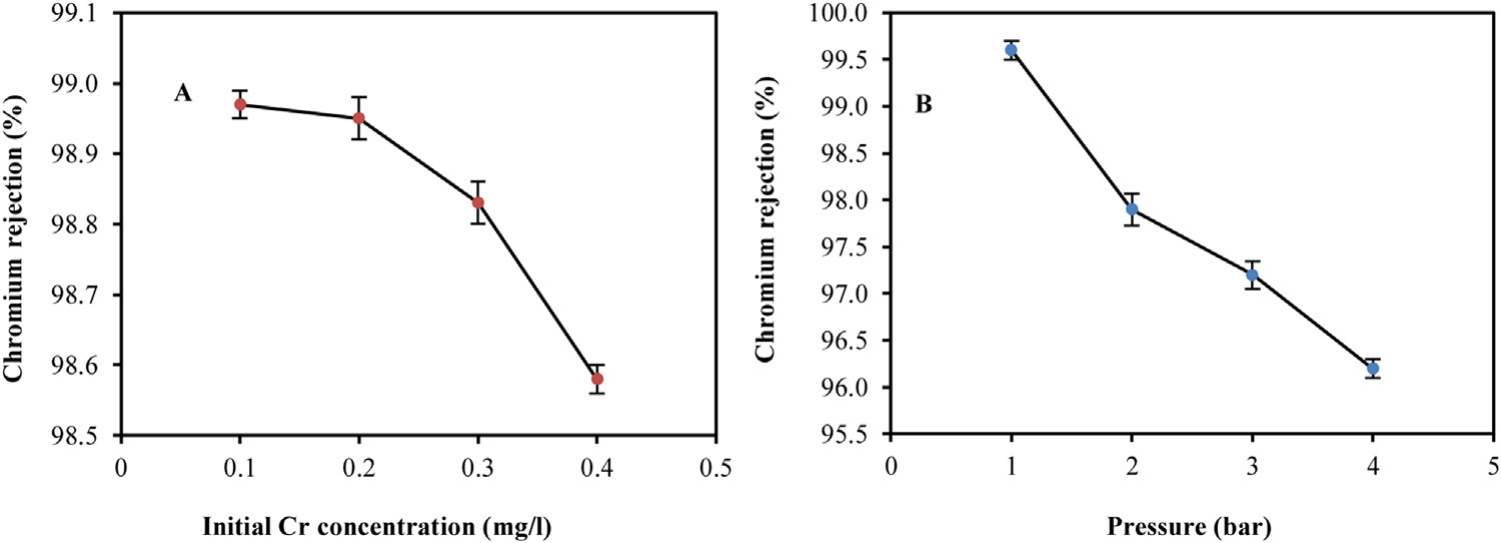
Effect of Cr concentration (mg/l) (A) and pressure (B) on the removal of chromium by fabricated hybrid system. The vertical lines represent the standard error.

Fig. 2A shows the efficiency percentage diagram of chromium removal as compared to oral solution concentrations at 0.1, 0.2, 0.3, and 0.4 mg/l concentrations. Keeping the pressure constant at 1 bar and a pH of 6.3 at this stage, the concentration variation effect on Cr*(VI)* removal was investigated. As can be seen, the efficiency has been equal to 98.97% at a concentration of 0.1 mg/l of chromium, which has decreased to 98.58% by increasing the concentration to 0.4 mg/l. Therefore, the removal rate decreases with increasing the initial Cr concentration. Thus, one can state that an inhomogeneous layer is gradually created in the inner wall of the membrane surface by passing the initial feed solution from the nanofilter membrane surface. By increasing the concentration of chromium in the feed tank and its passage through the nanofilter, the thickness of this layer increases quickly, leading to concentration polarization, which limits the passing flow, and thus, reduces the efficiency. In research by Muthukrishnan et al. [1] and Hafiane et al. [2], the efficiency of removal of hexavalent chromium from water by the nanofiltration membrane has been examined with the results similar to this study. They found that with increasing concentrations of Cr*(VI)*, the removal efficiency will be reduced. The results are consistent with the study of researchers such as Bruggen and Vandecasteele [3].

#### The effect of pressure on the efficiency of chromium (VI) removal

Fig. 2B shows the chromium removal from water at the pressures of 1, 2, 3 and 4 bar. As can be seen, at pressure of 4 bar, the removal efficiency is 96.21%, which increases up to 99.6% by reducing the pressure to 1 bar. One can conclude that by increasing the pressure at an input concentration, the amount of water passing through the fine pores of the membrane increases, and consequently, more chromium ions will remain on the membrane. This causes the phenomenon of polymerization to occur, increasing the membrane resistance. As a result, the concentration of chromium output at a certain concentrations decreases with increasing pressure. In a research conducted by the Ren et al. [4], it was found that the effects of pressures applied at pressures higher than 4 bar to remove Cr*(VI)* is insignificant. Therefore, the results obtained are consistent with the reports of the above researchers.

### The effect of pH on the efficiency of chromium (VI) removal

**Fig. 3:**
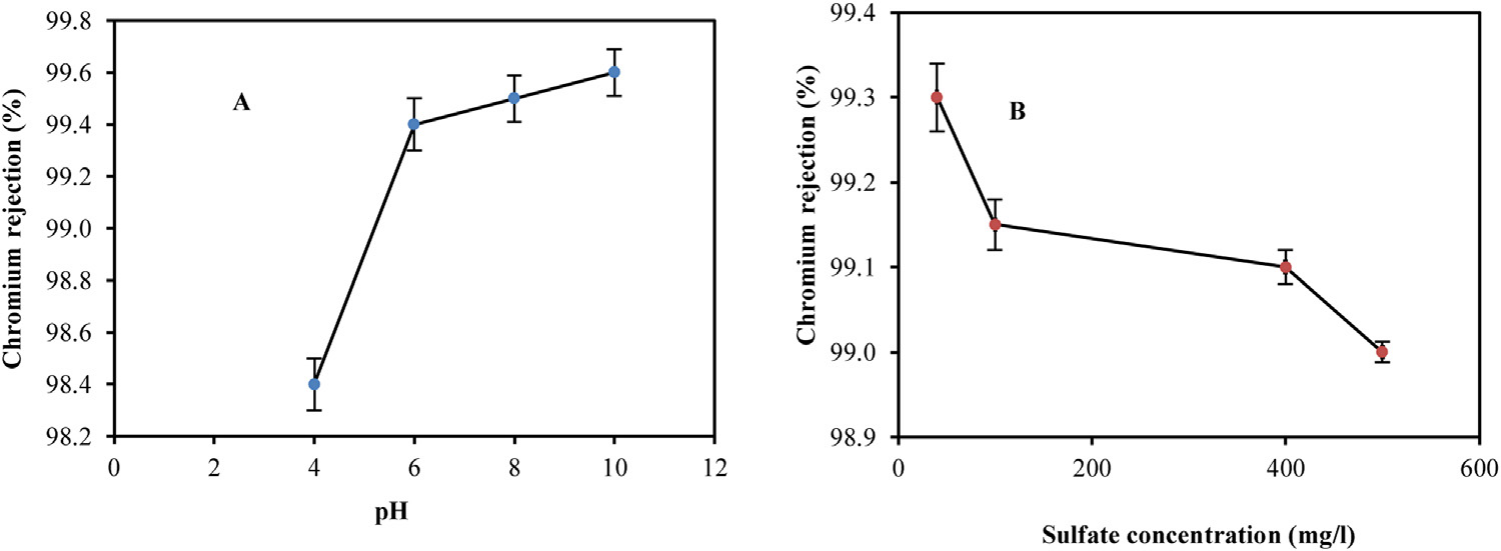
The effect of pH (A) and sulfate concentration (B) on the removal of hexavalent chromium by fabricated hybrid system. The vertical lines represent the standard error.

As shown in Fig. 3A, a basic pH increases the removal rate of chromium so that the highest removal rate was obtained at pH 10. According to the results, with increasing pH from 4 to 10, the removal percentage of Cr*(VI)* has increased from about 98.4% to over 99.6%. A reason for chromium removal efficiency increase due to increasing pH is the formation of bivalent chromium ($Cr\;O_{4}^{-2}$/ ${Cr}_{2}\;O_{7}^{-2}$) ions. These ions are usually found in pH over 7. Basically, hexavalent chromium appears as chromic acid in highly acidic pH(s), which are immediately converted into acidic hydrogen chromate (HCr$O_{4}^{-}$) up to a pH equivalent to 6.5. The divalent ion concentration of dichromate varies depending on the input concentration and pH conditions. The concentration of dichromate ions decreases in the presence of elevated levels of chromate ion and increased pH. Chromic acid breaks down under alkaline conditions and increases the bivalent chromium ions. These results are consistent with the results from the researches conducted by Muthukrishnan et al. [1], Hafiane et al. [2], and Barikbin [5].

### The effect of sulfate concentration on the efficiency of Cr removal

According to Fig. 3B, at pressure of 1 bar, Cr concentration of 0.4 mg/l, and pH of 6.3, concentration changes on Cr removal was evaluated. As can be seen, the efficiency rate has been 99.3% at a concentration of 40 mg/l of sulfate, which has reduced to 99% by increasing the concentration to 500 mg/l. Increasing the concentration of sulfate has led to a reduction in the efficiency of Cr*(VI)* removal. This phenomenon can be due to negatively charged membrane, electrostatic force, and ions stripping by the membrane, which has negatively stripped chromate ions, leading to increased hexavalent chromium removal rate. However, by increasing the ionic concentration of the solution, the stripping of ions usually reduces and chromate ions cross the membrane due to concentration polarization, which decreases the removal rate. The results correspond with the results reported by Yoon et al. [6] and Kouti et al. [7].

#### The removal efficiency of the hybrid system with actual water samples

**Fig. 4:**
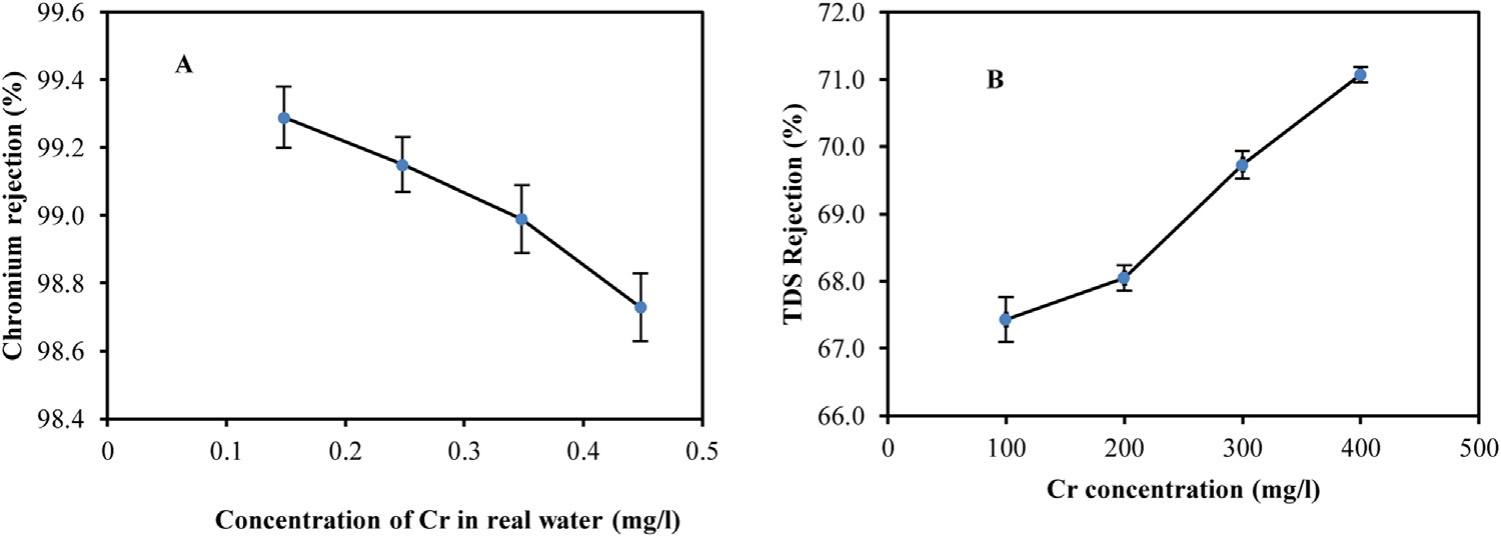
Removal of hexavalent chromium by the hybrid system in real water samples (A). The effect of initial concentrations of Cr in removal of TDS (B). The vertical lines represent the standard error.

Fig. 4A shows the chromium removal from actual water samples at the concentrations of 0.148, 0.248, 0.348, and 0.448 mg/l. At this stage, by keeping the pressure of 1 bar and pH of 8, the effect of concentration changes on Cr*(VI)* removal was examined. As can be seen, the removal efficiency has been equal to 99.29% at a 0.148 mg/l concentration of chromium (reducing concentration from 0.148 mg/l to 0.001 mg/l), which has reduced to 98.73% by increasing the concentration to 0.448 mg/l. Therefore, the rate of Cr*(VI)* removal reduces by increasing the initial concentration. We can state that when the concentration of hexavalent chromium ions increases in the feed solution, the accumulation of ions increased on the membrane surface due to reduced electrostatic repulsion. As the presence of chromium ions in real water intensifies this state, leading to increased osmotic pressure, this will ultimately reduce the efficiency of chromium removal in the nanofilter membrane. These results are also consistent with the results of researchers such as Vandecasteele and Bruggen [3].

#### The effect of removal efficiency of TDS at different concentrations of chromium

Fig. 4B shows the removal efficiency of TDS at concentrations of Cr in the range of 0.1-0.4 mg/l at pH of 6.3, pressure 1 bar, and ambient temperature. As seen, with increasing concentrations of Cr in the feed solution, the TDS removal efficiency rate increases. At a concentration of 0.1 mg/l of chromium, the efficiency rate of TDS removal has been 67.43%, which has increased to 71.07% by increasing the concentration to 0.4 mg/l. The presence of sulfate, magnesium, sodium, and calcium ions increases the TDS in the water. These ions accumulate on the membrane of the nanofilter by more than 50%. The increased hexavalent chromium and the presence of these ions can increase the amount of TDS in the water. This phenomenon occurs due to the reduced electrical layer on the membrane [8]. In other words, the hybrid system has a high ability to remove anions and cations found in water. Barikbin et al. [5] confirms an increase in the removal rate of TDS by increasing the concentration of chromium.

### Sulfate removal efficiency by the fabricated hybrid system

#### Effect of pressure on sulfate removal efficiency

**Fig. 5:**
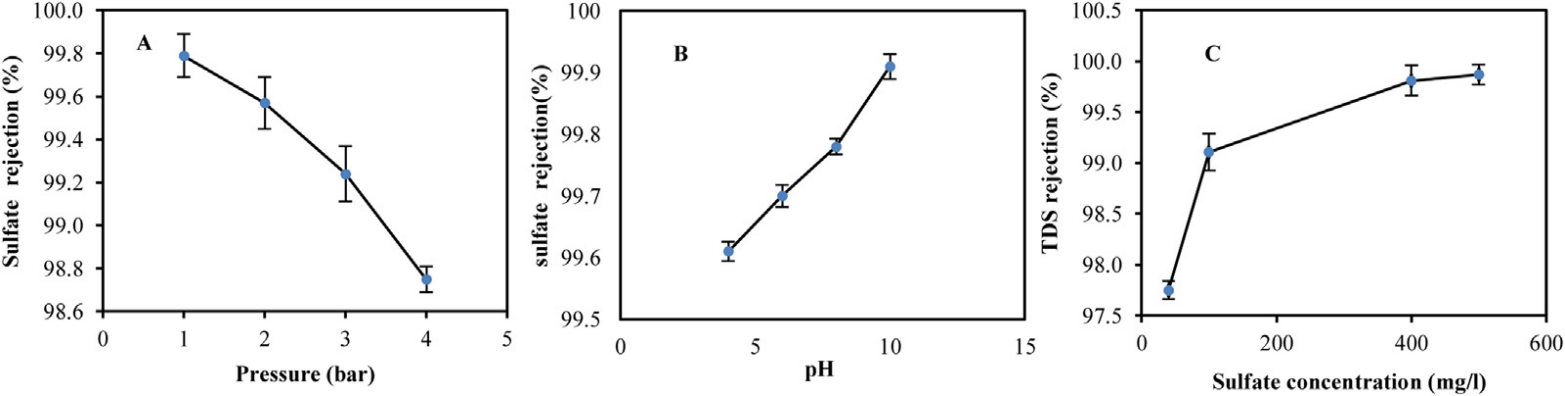
The effect of pressure (A) and pH (B) on the removal of sulfate onion by fabricated hybrid system. The effect of initial concentrations of sulfate in removal of TDS (C). The vertical lines represent the standard error.

At this stage, maintaining concentration of sulfate equivalent to 400 mg/l and pH 6.3, the pressure changes in sulfate removal were investigated (Fig. 5A). As can be seen, at the pressure of 1 bar, the removal efficiency is equivalent to 99.99% (reducing concentration from 400 mg/l to 0.04 mg/l) and decreases by 98.75% by increasing the pressure to 4 bar. The standard of sulfate in drinking water is 500 mg/l. At a pressurization of 4 bar, the concentration of soluble materials in a very close to the membrane increases significantly, therefore, due to the increase in surface pressure of the membrane, the water-soluble cations also have a low hydration energy, from the surface of the membrane of the nanofiltration run away. Also, given the fact that the water load should be neutral behind the nanofilter membrane, with the escape of cations, anions, most of which are ion sulfate, pass through the surface of the nanofilter membrane. As a result, with 4 times the pressure, the efficiency decreases [9].

#### Effect of pH on sulfate removal efficiency

The results (Fig. 5B) show that by increasing the pH from 4 to 10, the percentage of removal of sulfate has been improved from 99.61% up to 91.99% (concentration of 400 mg/l of sulfate, pH of 10, and pressure of 1 bar). Visser et al. investigated the sulfate removal by NF membranes and found that at neutral pH, the removal percentage of sulfate was higher than 99.9%, but at lower pH, it is less than 20% [10].

#### Removal efficiency of TDS at various concentrations of sulfate

Fig. 5C shows the removal efficiency of TDS in sulfate concentrations ranging from 40 to 500 mg/l. As can be seen, the percentage of removal efficiency of TDS increases with increasing concentrations of sulfate in the feed solution (pressure 1 bar, ambient temperature, and pH 6.3). At sulfate concentration of 40 mg/l, the TDS elimination efficiency was 97.75% and increased by 99.87% as the concentration increased to 500 mg/l. The presence of sulfate ions increases the TDS in water [7].

## Conclusion

In this study, we investigated the performance of a hybrid pilot of nanofiltration and microfiltration to remove hexavalent chromium from water. The results suggest that increasing sulfate concentration in the feed solution from 40 to 500 mg/l, the chromium removal reduces. The pH of 10 and a pressure of 1 bar are the best conditions for removal of hexavalent chromium and sulfate by the removal efficiency of 99.6%. Thus, the alkaline pH is the most optimal conditions for the removal of hexavalent chromium and sulfate. Also, under artificial conditions, when there is only hexavalent chromium in the feed tank that is passed through the hybrid pilot, the hexavalent chromium removal rate decreases by increasing the concentration. According to the findings of the study, the hybrid pilot made is able to reduce the chromium and sulfate to the levels under the standard.

## Additional information

Heavy metals have numerous uses in chemical and engineering industries [[11], [12], [13], [14]]. They have a lot of toxic effects on the human and environment [[15], [16], [17], [18]]. Chromium is found in trivalent and hexavalent forms in the aqueous systems often as chemical complex. The distribution of compounds containing hexavalent and trivalent chromium depends on the oxidation-reduction potential, pH, the presence of oxidizing and reducing compounds, the redox kinetics, formation of Cr^3^ complexes or insoluble Cr^3^ salts, and the total concentration of chromium [19]. The hexavalent chromium is more toxic than the trivalent chromium due to its high toxicity in water and rapid permeability through the biological membranes [20]. The toxicity rate of hexavalent chromium is 500 times of the trivalent chromium, which compounds include chromate, bichromate, and chromic acid [21]. This element can cause various health problems such as skin rashes, allergic reactions, lung cancer, and damage to the kidneys and liver [22]. If the concentration of chromium in the body reaches 0.1 mg/l, it can eventually lead to death. The risk-based drinking water standard is defined from of 0.05 mg/l for Cr*(VI)* according to World Health Organization standards [23]. The main sources of chromium arise from washing in plating industry, leather industry, and wood preservatives and pigments industries [24]. A wide range of technologies to remove hexavalent chromium and another heavy metal from water have been studied, including nanoporous materials [[25], [26], [27], [28]] ion exchange [29], absorption [30], deposition [31], membrane-based separation [32], and chemical complexes [33]. The sedimentation process is preferred in most cases due to its relatively simple purification nature. However, the whole chromium is discharged in the process as a hazardous sludge.

Hafiani et al. studied the removal of hexavalent chromium at different pH(s) [2]. Taleb-Ahmed and et al. [34] studied the behavioral effects of the physico-chemical properties of chromium and nanofiltration. In recent years, the membrane-based processes have become highly important around the world to achieve an effective separation method, which play a vital role in purifying water supplies to provide drinking water [35]. On the other hand, other operating costs of membrane-based methods are relatively lower compared to other methods [36]. Nanofiltration is a very complex process, depending on the surface micro-hydrodynamic events occurring on the surface and in the nanopores of the membrane. NF membranes have pores with a size smaller than 1 nm and a molecular weight cut-off of less than 300 to 500 Da [37]. They are capable of removing material with 0.01 to 0.001 μms in size. Their separation mechanisms include screening, dispersing the solution, and propulsion [38]. NF membranes, usually negatively charged, are covered with a selective layer of ˜ 1 μm thickness on a highly porous membrane layer that all the transmission properties are made through penetration and Donnan equilibrium [39]. Many researchers have studied the effect of physicochemical behavior [35] and pH [40] in the NF. Their studies have shown that the disposal rate depends on the ionic strength and water pH (up to 80% at pH 8). A lot of research has been done on removing chromium at concentration of 5–2000 mg/l [39], which are higher than levels found in drinking water supplies. Therefore, there is a lack of information about the removal of chromium by NFs at the levels in the drinking water. Thus, this study was mainly designed to examine the NF behavior in removing Cr*(VI)* from drinking water. To this end, we studied the effects of important effective factors, namely, pressure, pH, concentration of hexavalent chromium, total dissolved solids, and the concentration of sulfate in removing hexavalent chromium by using a hybrid system (sand filter, carbon filter, microfilter, and nanofilter). Furthermore, removal of sulfate anion was investigated by the fabricated hybrid system. Samadi et al. [41] studied the efficiency of reducing nickel from aqueous environments by using carbon nanotubes. The highest removal efficiency was 82.5%. At another study, novel γ-alumina nanoparticles and multiwalled carbon nanotubes were synthesized for the rapid removal of the nickel *(II)* from the solvent phase [42]. Percentage removal using carbon nanotubes and novel γ-alumina nanoparticle was 87.65% and 99.41%, respectively under optimum conditions.
